# In Vitro Antifungal Activity of *Peltophorum dubium* (Spreng.) Taub. extracts against *Aspergillus flavus*

**DOI:** 10.3390/plants9040438

**Published:** 2020-04-02

**Authors:** Lucía S. Di Ciaccio, Alejandra V. Catalano, Paula G. López, Dante Rojas, Diego Cristos, Renée H. Fortunato, Adriana E. Salvat

**Affiliations:** 1 Instituto de Patobiología Veterinaria, (IPvet), CICVyA, Instituto Nacional de Tecnología Agropecuaria, Hurlingham 1686, Prov. de Buenos Aires, Argentina; diciaccio.lucia@inta.gob.ar; 2 Consejo Nacional de Investigaciones Científicas y Técnicas—CONICET, Ciudad Autónoma de Buenos Aires 1425, Argentina; vcatalano@ffyb.uba.ar (A.V.C.); plopez@ffyb.uba.ar (P.G.L.); fortunato.renee@inta.gob.ar (R.H.F.); 3 Facultad de Farmacia y Bioquímica, Cátedra de Farmacognosia, Universidad de Buenos Aires, Ciudad Autónoma de Buenos Aires 1113, Argentina; 4 Instituto de Tecnología de Alimentos, Instituto Nacional de Tecnología Agropecuaria, Hurlingham 1686, Prov. de Buenos Aires, Argentina; rojas.dante@inta.gob.ar (D.R.); cristos.diego@inta.gob.ar (D.C.); 5 Instituto de Recursos Biológicos, CIRN, Instituto Nacional de Tecnología Agropecuaria, Hurlingham 1686, Prov. de Buenos Aires, Argentina; 6 Facultad de Agronomía y Ciencias Agroalimentarias, Universidad de Morón, Morón 1708, Prov. de Buenos Aires, Argentina

**Keywords:** *Aspergillus flavus*, flavonoids, plant bioactivity, evans blue, active extract characterization

## Abstract

*Aspergillus flavus* is a filamentous, saprophytic fungus, whose colonization occurs mainly in cereal grains and oilseeds once harvested. Under certain conditions, it could produce mycotoxins called aflatoxins, known as powerful human liver carcinogens. The aim of the present study was to describe the antifungal activity of extracts of *Peltophorum dubium*, a species from northern Argentina (Oriental Chaco), against *A. flavus*. The antifungal activities of different collection sites are reported. The extracts exhibited a minimum inhibitory concentration of 125 µg/mL, and the differences between the treatments and the inoculum control were 11 mm of *P. dubium A* and 10 mm of *P. dubium F* in colony growth. Moreover, hyphae treated with the extracts stained blue with Evans blue showed alterations in the membrane and/or cell wall, allowing the dye income. Bio-guided fractionation, High Performance Liquid Chromatography diode array ultraviolet/visible (HPLC UV/VIS DAD), and Ultra-High Performance Liquid Chromatography Electrospray Ionization Mass Spectrometry (UPLC ESI-MS) analyses were conducted to characterize the extracts and their active fractions. The HPLC UV/VIS DAD analysis allowed the determination of the presence of flavonoids (flavonols and flavones), coumarins, terpenes, and steroids. UPLC ESI/MS analysis of active fractions revealed the presence of Kaempferol, Apigenin, Naringenin, Chrysin and Daidzein.

## 1. Introduction

Millions of tons of cereals suffer some kind of deterioration, among others, due to the action of fungi that cause serious economic damage due to yield losses and/or alterations in the quality of the grains [1,2,3]. Among fungi that produce losses in cereal quality, the genus *Aspergillus* has the ability to grow in different substrates and under a wide range of conditions, especially during storage [4]. When the grain is harvested, it contains an abundant spore load that comes from the field, which is maintained during transportation and storage. If storage conditions are not adequate, grain deterioration and mycotoxin formation [5], such as aflatoxins (AF), may occur. This group of substances, produced by the fungi *Aspergillus flavus* and *Aspergillus parasiticus*, are known for their high toxicity for both humans and animals [6,7] and can cause functional, biochemical, or morphological alteration, generating teratogenesis and mutagenesis, making the liver the mainly affected organ. They also induce the formation of tumors in the kidney, colon, and lung [8,9]. Unfortunately, these toxins appear very frequently and in varying concentrations in cereals, causing losses in commercialization and exportation.

*Peltophorum dubium* Spreng. Taub. (Caesalpinioideae, Fabaceae = Leguminosae) is a tree from the riverbanks in the south of Brazil, the northeast of Argentina and Paraguay, and in the north of Uruguay on the Eastern Chaco region [10]. There is data that highlight the leaves and fruits with laxative, digestive, hepatoprotective, and astringent effects [11,12,13,14]. Regarding the methanolic extracts of the branches, antimicrobial activity was recorded against a strain of *Staphylococcus aureus* [15]. On the other hand, the seeds have shown insecticidal activity against *Anagasta kuehniella* (Lepidoptera: Pyralidae) [16], and a trypsin inhibitor that induces cell death in human leukemia [17] was also isolated. Moreover, Reference [18] managed to isolate C-glucoside benzoic acid derivative, a new compound, from the leaves with antioxidant activity. It should be noted that other species of the genus *Peltophorum*, such as *P. Pterocarpus,* were studied for their antibacterial and antifungal activity [19,20] and also as an antioxidant [21,22], while from the *P. Vogelianum* species, several compounds were isolated, from which peltophorumyl β-D glucupyranoside with antibacterial properties stands out [23].

The aim of this work is to evaluate the antifungal activity and related active principles of the leaf extracts of *Peltophorum dubium* from two very different phytogeographic regions, collected in different years, against a strain of *A. flavus,* a producer of aflatoxins.

## 2. Results

According to the obtained results by the microdilution method of the 16 methanolic studied extracts of *P. dubium*, only 2 exhibited antifungal activity against *A. flavus*, both from leaves and with a minimum inhibitory concentration (MIC) of 125 µg/mL. They were called *P. dubium* (*A*), from the province of Chaco, and *P. dubium* (*F*), from the province of Buenos Aires. The collections were made in different years, different phytogeographic areas, and in different phenological states, and, in all cases, the antifungal activity of these extracts remained. 

**Figure 1:**
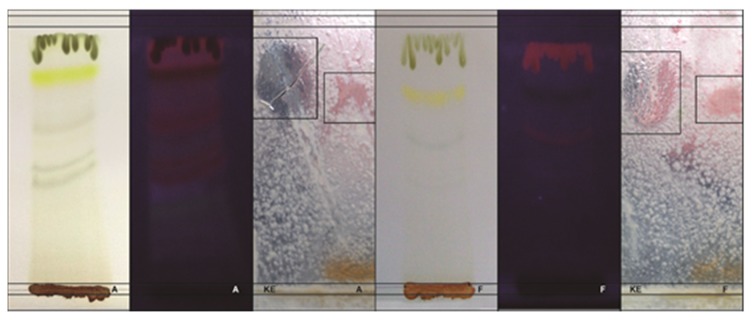
Contact bioautography: Extracts A and F of *P. dubium* against *A. flavus*. From left to right: (*A*), thin layer chromatography (TLC) of *P. dubium* leaf extract (visible light); UV at 366 nm and contact bioautography. (*F*) TLC of *P. dubium* leaf extract (visible light); UV at 366 nm and contact bioautography.

With reference to the contact bioautography technique, it was determined that the mobile phase Chloroform:Methanol (CHCl_3_:MeOH) (9:1) showed better separation of the compounds, and in both extracts, the inhibition zone was at Rf = 0.85. Various orange fluorescent bands were displayed in ultraviolet light (366 nm), including the band responsible for the inhibitory effect that showed a faint fluorescence; however, this same band in visible light exhibited an intense yellow color (Figure 1).

**Figure 2:**
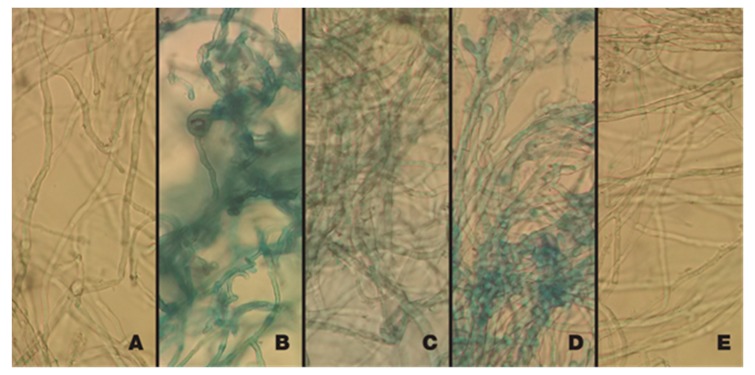
Staining with Evans blue: Hyphae of *A. flavus* exposed to extracts *A* and *F* of *P. dubium*. (**A**) Control. (**B**) Ke. (**C**) *P. dubium* leaves extract (*A*). (**D**) *P. dubium* leaves extract *(F)*. (**E**) DMSO: MeOH (9:1).

As for the staining technique with Evans blue dye, the hyphae of *A. flavus*, which were treated with the active extracts *A* and *F* at their respective MICs, were stained blue and also showed a noticeable change in their morphology, as is the case with the antifungal Ketoconazole (size increase, widening of the hyphae). In contrast, the control hyphae and those exposed to the Dimethylsulfoxide (DMSO):MeOH (9:1) solution maintained their natural color (translucent) and their original form (Figure 2).

**Figure 3:**
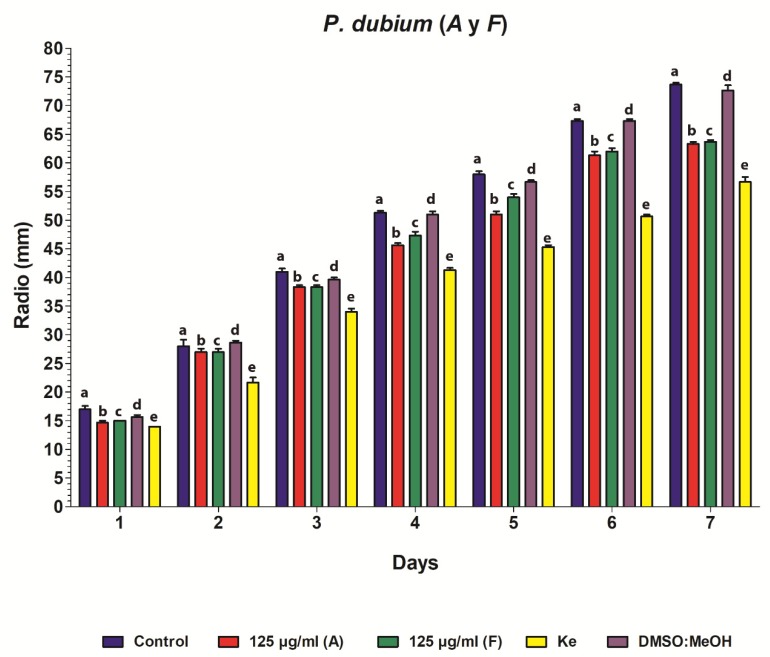
Hyphal radial growth test. The obtained data in this test was analyzed by analysis of variance (ANOVA), followed by the Bonferroni post-test. The analysis was expressed as the mean (*x*) ± SD. Data are shown as the average values of fungal growth rate for each treatment. Different letters (a, b, c, d, e) indicate significant differences (*p* < 0.05) among treatments. (Control: blue, *P. dubium A*: red, *P. dubium F*: green, Ke: yellow, DMSO:MeOH: violet).

On the other hand, in the radial mycelium growth test, when *A. flavus* was treated with extracts *A* and *F* of *P. dubium*, a delay in the development of the mycelium of 5 mm and 4 mm was observed, respectively, from the fourth day, regarding the control. At the end of this test, the difference was greater, between 10 and11 mm. An important fact was confirmed that the dilution solvent of the extract, DMSO: MeOH (9:1), did not affect the bioactivity since its inhibition was minimal, so that the antifungal activity would be given only by the extracts. In order to define the extracts as fungicides, they must completely inhibit the development of fungi; however, the action of the studied extracts showed an inhibition in the development of fungi of less than 99.99%, proving to be of the fungistatic type (Figure 3).

In both methanolic extracts, *A* and *F*, the activity was present in the fractions obtained with the dichloromethane solvent (DCM) (fractions 7, 8, and 9). The chemical characterization of the methanol extracts *A* and *F* and their active fractions were performed by high performance liquid chromatography diode array ultraviolet/visible (HPLC UV/VIS DAD) and thin layer chromatography (TLC) analysis with different spray reagents used for the detection of compounds. In *A* and *F* extracts and their active fractions, it was determined that there were no qualitative differences. The presence of flavonoids, flavones, and flavonols, present in a majority proportion, was detected. Additionally, coumarins and less polar compounds, such as terpenes and steroids, were detected [24]. 

The Ultra-High Performance Liquid Chromatography Electrospray Ionization Mass Spectrometry (UPLC ESI/MS) showed the presence of flavonoids Kaempferol, Naringenin, and Chrysin in fraction 7, Apigenin in fraction 8, and in fraction 9 Apigenin, Naringenin, and Daidzein. (Supplementary Materials).

## 3. Discussion

A great variety of diseases that affect plants can cause, on one hand, considerable economic losses for the farmer, due to the decrease of his production, and also it can originate controversies at the moment of commercialization, due to the demands of the buyer countries. This problem places plant health as a topic of great interest worldwide; in that regard, the maximum efforts should be devoted to prevention, because even if consumer’s health could be protected, there are still significant economic losses [25]. With the current level of knowledge, prevention strategies include strict control of environmental conditions during post-harvest management and, occasionally, the use of antifungal substances during critical periods in the crop and/or during storage or transportation.

Another aspect of significant importance is the implications on human and animal health that involve *A. flavus* in reference to the presence of mycotoxins and to the damages that occur in immunologically compromised patients. In the last few decades, due to the resistance of the use of fungicides [26], different investigations have been oriented and promoted, involving the study of plant extracts as an alternative for their control [27,28,29,30,31,32]. 

In our case, motivated by the bioactive characteristics present in *P. dubium* branches against bacteria [15], we consider it very interesting to evaluate its potential antifungal activity. It was possible to determine that the extracts of its leaves showed activity against the fungus *A. flavus*, with the particularity that this activity is conserved in both the collected material in the area where it is native (Chaqueña Region) and in the material of the area where it was introduced (Pampeana Region). Both active extracts, *A* and *F*, have the same Minimum Inhibitory Concentration (MIC)- and their behavior is similar. Its activity was evidenced when Evans blue staining was performed, since modifications or morphological changes in the hyphae were observed. Both extracts caused a widening of the cell wall and also alterations that could involve the membrane, allowing the entry of the dye. It is important to highlight that the modifications of this type were cited by Reference [33] from a series of investigations with plant derivatives, concluding that “the plant compounds collapse the cell wall and plasma membrane, penetrating through them, affecting several intracellular functions with the consequent alteration of normal mycelial growth”. Moreover, like other compounds extracted from plants, *P. dubium* possesses a fungistatic capacity, because it causes dilation in the growth of the mycelium of *A. flavus*, which is later maintained over time. This had not been previously described in this plant and is a new contribution.

The study of chromatographic profiles and spectroscopic characteristics of secondary metabolites present in extracts of plant origin with biological activity is essential to characterize them [34,35]. The characterization of the active plant extracts is the first step to perform the bio-guided fractionation for the activity and is also the reinsurance of the reproducibility of the activity. From this, the importance of characterizing the extracts arises, as was done in the UV-visible and MS analysis to standardize the *P. dubium* extract.

The determination of the presence of flavonoids as the possible compounds responsible for antifungal activity against *A. flavus* is an interesting discovery, since there are numerous studies that consider these and other flavonoids as responsible for various activities. Such is the case of antifungal and antiviral activity of flavone apigenin and its glycosides (7 and 4), which give resistance to infection in *Pyrus* [36]. In the case of flavonol Kaempferol, its glycosides, isolated from stems of *Dianthus caryophyllus*, show antifungal activity against *Fusarium oxysporum* [37]. Additionally, it is important to highlight the effect that flavonoids have on the protection of cereals against fungi and their relationship with the resistance of wheat and barley cultivars, as well as the decrease in the production of mycotoxins [38,39]. It is known that flavonoids fulfill important metabolic functions in the plant kingdom and their concentration varies depending on the species and environmental characteristics, etc. [40]. Although research on antifungal activity from plants has a development time, they are still an opportunity for the search for new antifungal agents. Plant extracts are complex matrices, so it is appropriate to emphasize the variation that can be observed and the criteria that must be taken into account when assessing antimicrobial activity. There are multiple variables that can generate influence on the results, such as climate conditions, in which the plants grow because it can cause a change in the concentration of their metabolites, the method of evaluation, which is generally adapted in each laboratory, the extraction solvent used, the microorganism to be studied, and the selected strain [41,42].

It is important to emphasize that, due to different factors, only a few classes of antifungal agents are currently available, being sometimes ineffective, since they have developed resistance or because they are too toxic for the host, causing unwanted side effects, and also because the appearance of fungi, called re-emerging, is known. These are some reasons we find ourselves in need of producing new antifungal agents [26,43].

We believe that it is necessary to improve systematically towards this kind of study, where it is revealed or known what type of bioactivity the plants possess, and oriented towards obtaining results that lead to useful applications, especially in areas such as medicine, agriculture, industry, etc., thus achieving the goal of adding value to plant diversity.

## 4. Materials and Methods

### 4.1. Plant Collection and Identification

The plant specimens that were used were collected and identified by R.H.F. The voucher of plants materials were deposited at BAB Herbarium (Instituto de Recursos Biológicos, CIRN, INTA): http://sciweb.nybg.org/Science2/IndexHerbariorum.asp.

Sixteen crude methanolic extracts of *P. dubium* (Spreng.) Taub. from two regions, at different periods, from different years and from various organs (leaves, fruits, branches, bark, and flowers), were studied. The selected collection areas were the Province of Chaco, Department of Bermejo, Isla del Cerrito, Argentina, and the town of San Isidro, in the province of Buenos Aires, Argentina.

### 4.2. Extraction of P. dubium

Each plant material was dried, finely ground, and extracted with MeOH (Merck) (10 g of dry plant material per 100 mL) at room temperature in total darkness for 48 h. The extracts were filtered, dried under reduced pressure at 40 °C, and weighed. These crude methanolic extracts were redissolved in MeOH at a concentration of 80 mg dry matter per mL. For extract conservation, 1 mL of each extract was diluted with 9 mL of dimethyl sulfoxide (DMSO; Biopack) until a final concentration of 8000 µg/mL. This solution was sterilized by passing through a 0.45 µm cellulose acetate membrane (Minisart, Sartorius). All extracts were kept in cryovials at −35 °C until analysis [44].

### 4.3. Microorganism, Media, and Solutions

#### 4.3.1. Microorganism

We worked with a strain of *A. flavus* that belongs to the collection of the Institute of Veterinary Pathobiology (IPvet) with recognized toxicogenic capacity, preserved in silica gel [45]. Cultures were prepared in tubes containing Papa Dextrose Agar (PDA), incubated at 28 °C for 7 days. Peptone water (1%) + Tween 80 was then added to each tube. The conidial suspension was filtered through a sterile gauze inside a glass jar. Conidia were counted with a Neubauer chamber and the suspension was then adjusted to a concentration ranging 1–3 × 10^4/5^ conidia per mL, depending on the assay to be performed [44].

#### 4.3.2. Media

Roswell Park Memorial Institute 1640 (RPMI 1640) synthetic medium (Gibco by Life Technologies, Grand Island, NY, USA) was used 1× with L-glutamine without phenol red, oxoid PDA medium (potato dextrose agar).

#### 4.3.3. Solutions

Resazurin was used as dye (0.01% aqueous solution) from Sigma-Aldrich (St. Louis, MO, USA). Ketoconazole (Ke) was used at a concentration of 2 µg/mL for the colorimetric assay for antifungal susceptibility testing. Evans blue staining and hyphal radial growth test at 50 µg/mL for contact autobiography were also used. All the solutions were sterilized using sterile filters with 0.45 µm cellulose acetate membrane and preserved in cryovials at −35 °C.

### 4.4. In Vitro Antifungal Activity of Methanolic Extract

#### 4.4.1. Microdilution Method: Minimum Inhibitory Concentration (MIC)

This assay was conducted following the procedure indicated in M38-A [46], with some modifications for antifungal activity [44]. This technique was conducted in sterile 96-well microplates Biofil (Guangzhou, China). Then, 200 µL of the MeOH: DMSO solutions (1:9) of the plant extracts were added to each of the wells in row A, and 100 µL of RPMI 1640 was added to the remaining wells (rows B–H). For preparing dilutions, 100 µL was removed from row A wells and added to wells in row B; the same procedure was repeated until reaching row H. The excess dilution (100 µL) of row H was discarded. Thus, the highest and lowest extract concentrations corresponded to wells in row A and in row H, respectively. Then, 100 µL of the inoculum, which contained between 1–3 × 10^4^ conidia per mL in all the wells, was added to all the wells, except for the RPMI 1640 medium control. Finally, 20 µL of Resazurin (Rz) was added to all the wells [47]. A number of wells in each plate were reserved for the control of RPMI 1640 sterility, inoculum viability, and the antifungal agent (Ke) at the afore-mentioned concentration and MeOH:DMSO (1:9) solvent effect. Microplates were incubated at 28 °C in the dark for 48 h. The color change in Rz from blue to pink indicated fungal growth. The MIC was considered the highest concentration (in µg of dry matter per mL of medium), at which no color change was detected. An extract was considered active at a MIC <500 µg/mL [48,49]. This assay was performed in triplicate.

#### 4.4.2. Contact Bioautography

The components of active extracts were separated by TLC. As the stationary phase, Silica gel 60 TLC plates (9 × 5 cm) were used and the mobile phase consisted of chloroform (methanol (9:1)) [50]. PDA was inoculated with 1–3 × 10^5^ conidia per mL of *A. flavus* and then placed in Petri dishes. Methanolic extracts were used at a concentration of 400 mg/mL and Ke positive control. Fluorescent bands in TLC were observed under UV light and placed on inoculated agar at 28 °C for 24 h. They were then removed and incubation continued for 24 h. Finally, inhibition zones were read and the Rf was calculated [51].

#### 4.4.3. Evans Blue Staining: Visualization of Hyphal Alterations

Spores of *A. flavus* were incubated in RPMI 1640 liquid medium at 28 °C for 24 h. Then, 1 mL of the medium containing the hyphae was transferred to 1.5 mL Eppendorf tubes. The active extracts at their respective MICs were individually added to the tubes; in addition, two tubes were included, one as positive control (Ke) and another one as inoculum. All the tubes were incubated at 28 °C for 24 h and then centrifuged; the culture media were removed and two drops of Evans blue (0.05%) were added for 5 min. Finally, the hyphae were washed with sterile distilled water to remove excess stain. The difference in staining was observed under light microscope at 400× and evaluated, following References [52,53].

#### 4.4.4. Hyphal Radial Growth Test

The antifungal activity of the extracts was evaluated using this test, and 7-day culture of *A. flavus*. Discs (5 mm) of the culture were taken and placed in the center of Petri dishes containing 20 mL of PDA with the active extracts at their respective MICs. In addition, the respective controls were performed, corresponding to the untreated inoculum, the inoculum with a Ke, and the control of the solvent extract. All the plates were incubated at 28 °C for 7 days. Colony radio (mm) was measured every day and treatment efficiency was evaluated [54,55]. This assay was performed in triplicate. 

### 4.5. Statistical Analysis

Data on fungal colony growth were analyzed by analysis of variance, followed by the Bonferroni post-test. The analysis was expressed as mean SD and differences were statistically significant at *p* < 0.05.

### 4.6. Characterization of P. dubium Extracts

#### 4.6.1. Isolation of Bioactive Fractions

Crude methanolic extracts (1 mL) were also fractionated by silica gel 60 (0.063–0.200 mm), column 20 cm length × 1 cm internal diameter, and eluted with 5 mL hexane (Hx), 5 mL dichloromethane (DCM), 5 mL ethyl acetate (EtOAc), and 5 mL methanol (MeOH) (fractions 1–20) [56]. All extracts and fractions were tested by the microdilution method and active samples analyzed by UPLC ESI-MS.

#### 4.6.2. Thin Layer Chromatography (TLC) Analysis

Methanol extracts and the active fractions were analyzed with the following chromatographic systems:

System 1: Stationary Phase Silica gel 60 F_254_; Movil Phase: Hexane: Ethyl Acetate (5:5); Detection I: UV 366 nm, Detection II: Natural Product Reagent/ UV 366 nm.

System 2: Stationary Phase: Silica gel 60 F_254_; Movil Phase: Toluene: Ethyl Acetate: Formic Acid (5:4:1); Detection I: UV 366 nm; Detection II: Natural Product Reagent/ UV 366 nm.

System 3: Stationary Phase: Silica gel 60 F_254_; Movil Phase: Toluene: Ethyl Acetate: Formic Acid (6:4:1); Detection I: UV 366 nm; Detection II: Natural Product Reagent/ UV 366 nm.

System 4: Stationary Phase: Silica gel 60 F_254_; Movil Phase: Hexane: Ethyl Acetate (5:5).

Detection: Vanillin sulfuric reagent. 

The chromatographic systems used are based on Reference [57].

#### 4.6.3. High Performance Liquid Chromatography Diode Array Ultraviolet/Visible (HPLC UV/VIS DAD) Analysis

A Varian ProStar^®^ High Performance Liquid Chromatography device with a UV/visible diode array detector (UV/VIS-DAD) was used, with a Phenomex Luna^®^ RP 18 analytical column (150 mm × 4.6 nm; 5 µm) and a binary mobile phase formed by Solvent A: water: acetic acid (98:2) and Solvent B: methanol: acetic acid (98:2). It was then eluted in the lineal gradient: 85% to 60% A in B (30 min), 60% to 25% A in B (10 min), and 25% to 15% A in B (20 min). The UV/visible detection was conducted between 200 nm and 800 nm. The flow rate was 1 mL/min. The ultraviolet-visible spectra of the compounds were compared with the description of Reference [58].

#### 4.6.4. Ultra-High Performance Liquid Chromatography Electrospray Ionization Mass Spectrometry (UPLC ESI-MS) analysis

A waters acquity ultra performance liquid chromatography (UPLC) apparatus equipped with a single quadrupole mass detector using XBridge BEH C18 2.5 Um 2.1 × 150 mm column, 0.1% acetic acid in water: methanol at the following gradient: (90:10)–(90:10) 0–0.5 min, (90:10)–(60:40) 0.5–3 min, (60:40)–(0:100) 3–9 min, (0:100)–(0:100) 9–11 min, (0:100)–(90:10) 11–12 min, (90:10)–(90:10) 12–15 min as the mobile phase and 0.25 mL/min flow throughout the chromatography run. The mass-spectrometer acquisition settings were: ESI negative and positive in FULL Scan Mode, in the mass range of 100–800 dalton.

The identification of compounds was performed using UPLC ESI-MS, comparing with the standard compounds as a pick of an active fraction. Retention time and spectrums were used as identification criteria.

#### 4.6.5. Compounds

Apigenin, Quercetin, Naringenin, Chrysin, Genistein, Daidzein, Kaempferol, and Rutin from Sigma–Aldrich^®^ HPLC solvents were purchase from Merck KGaA, Darmstadt, Germany.

## 5. Conclusions

The observed susceptibility allowed *P. dubium* to be included among the few species registered with this activity. In future studies, extracts of *P. dubium* and its active compounds could be part of new antifungal products to be applied in grains or at their storage site.

## Supplementary Materials

The following are available online at https://www.mdpi.com/2223-7747/9/4/438/s1, Figure S1: Spectrum Fraction DCM 7 and Chrysin, Figure S2: Spectrum Fraction DCM 7 and Kaempferol, Figure S3: Spectrum Fraction DCM 7 and Naringenin, Figure S4: Spectrum Fraction DCM 8 and Apigenin, Figure S5: Spectrum Fraction DCM 9 and Apigenin, Figure S6: Spectrum Fraction DCM 9 and Daidzein, Figure S7: Spectrum Fraction DCM 9 and Naringenin.
