# Supplementary figures and images for: In Vitro Antifungal Activity of Peltophorum dubium (Spreng.) Taub. extracts against Aspergillus flavus
Source: Plants (Basel). 2020 Apr 2;9(4):438. doi: 10.3390/plants9040438 (PMC7238424; doi:10.3390/plants9040438)

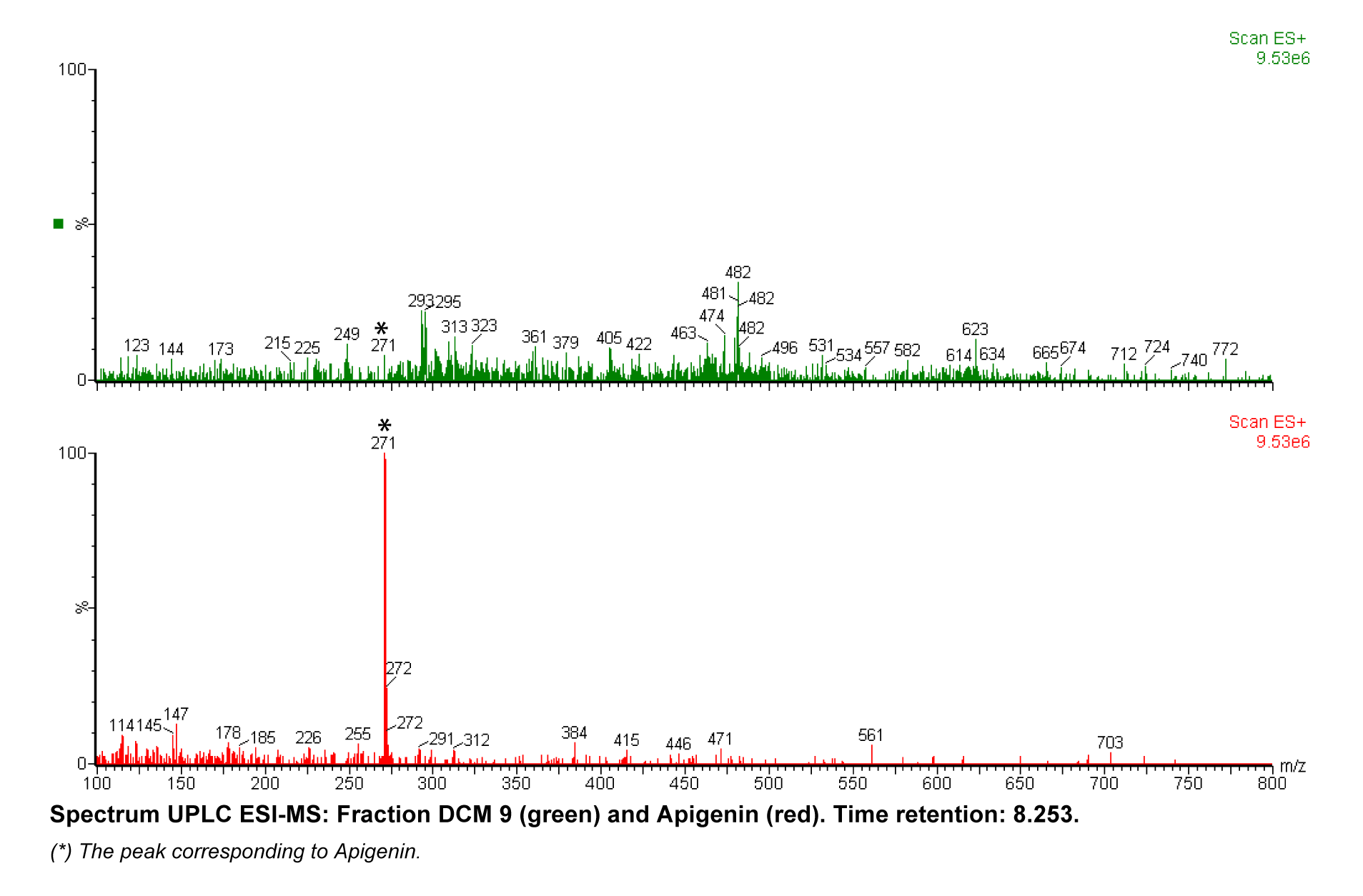

Supplement: Supplementary file 1 [file plants-09-00438-s001.zip › fraction dcm 9 and standard_Spectrum Fraction DCM 9 and Apigenin.tif]

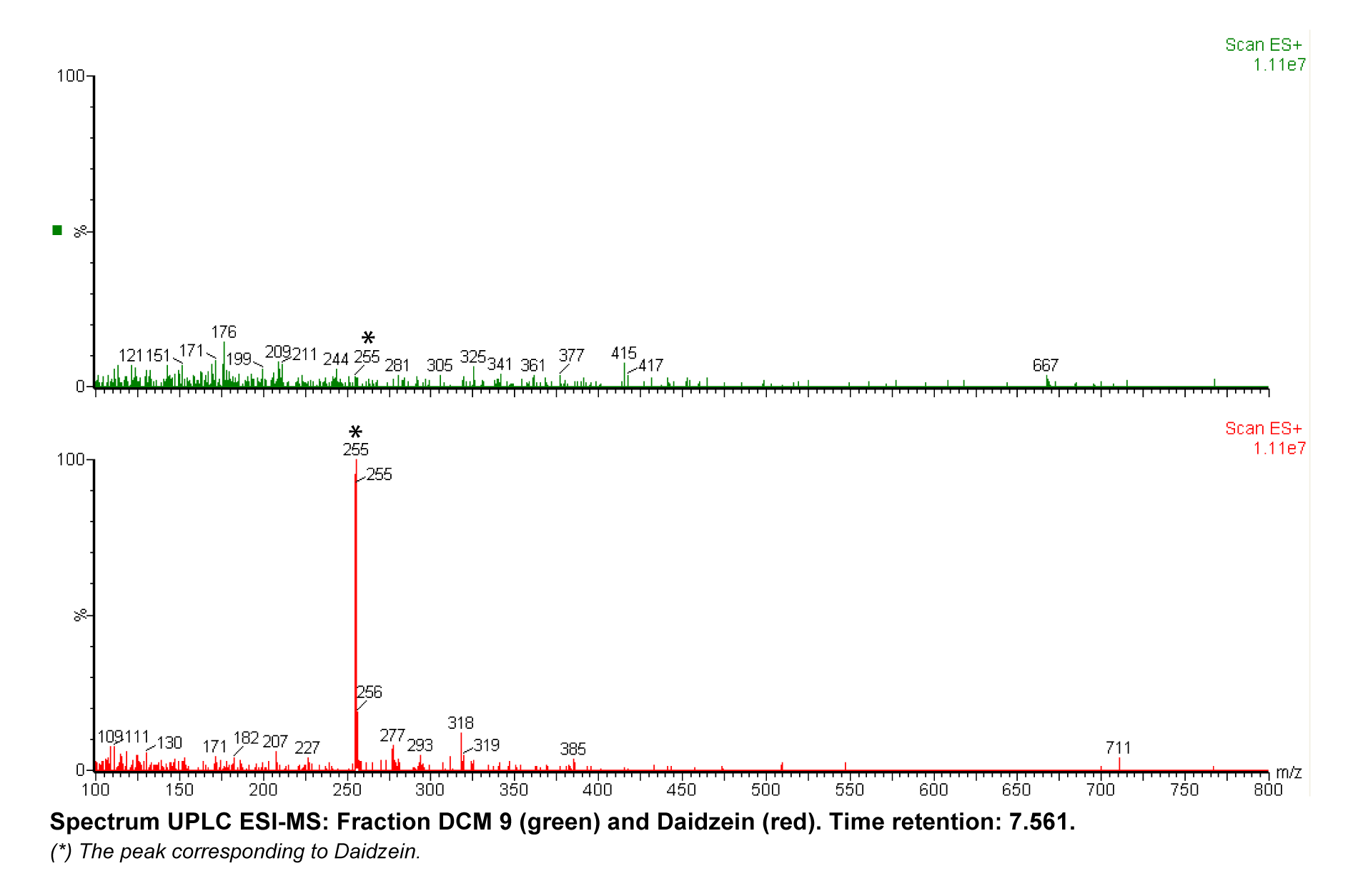

Supplement: Supplementary file 1 [file plants-09-00438-s001.zip › fraction dcm 9 and standard_Spectrum Fraction DCM 9 and Daidzein .tif]

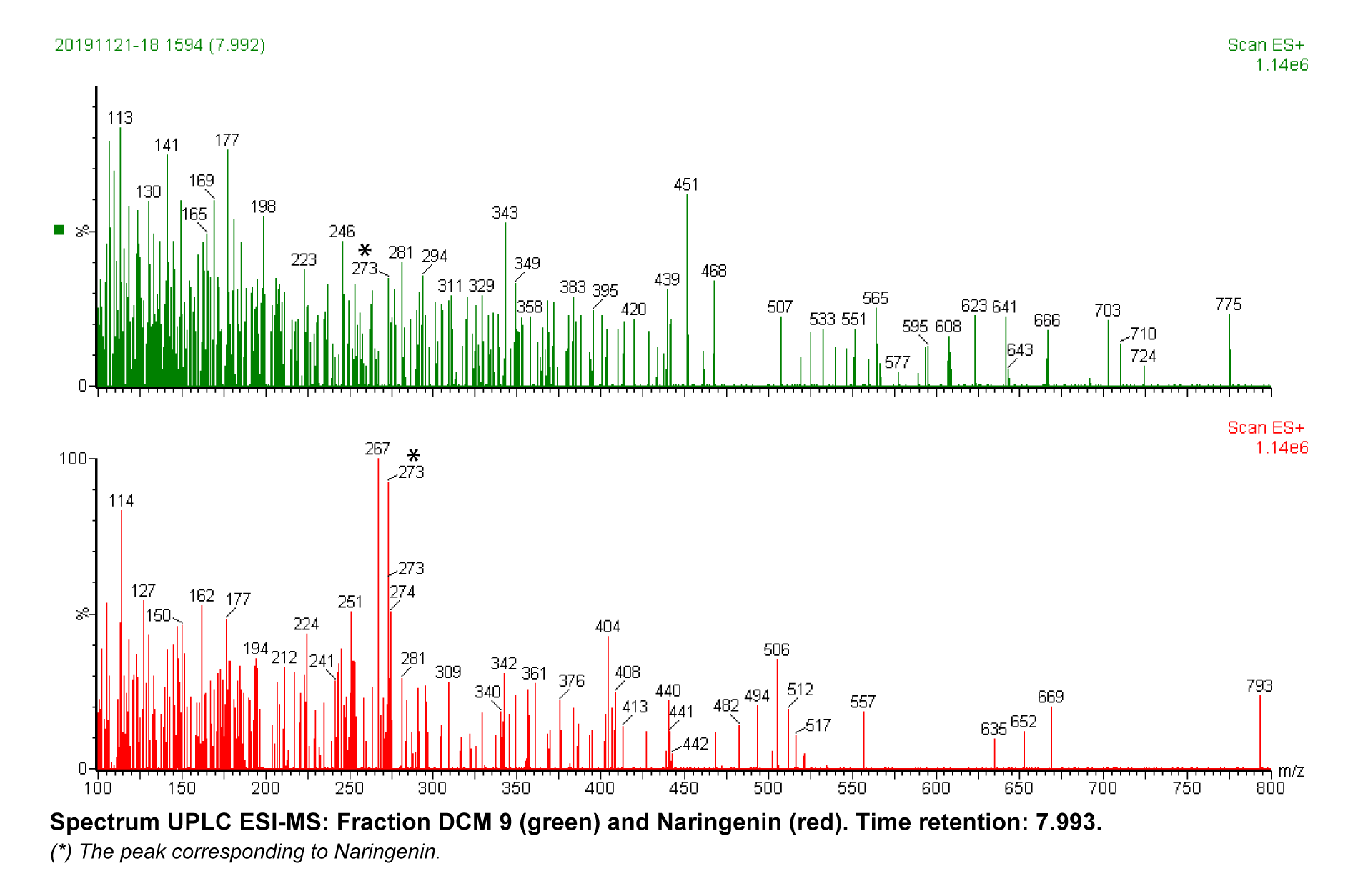

Supplement: Supplementary file 1 [file plants-09-00438-s001.zip › fraction dcm 9 and standard_Spectrum Fraction DCM 9 and Naringenin.tif]

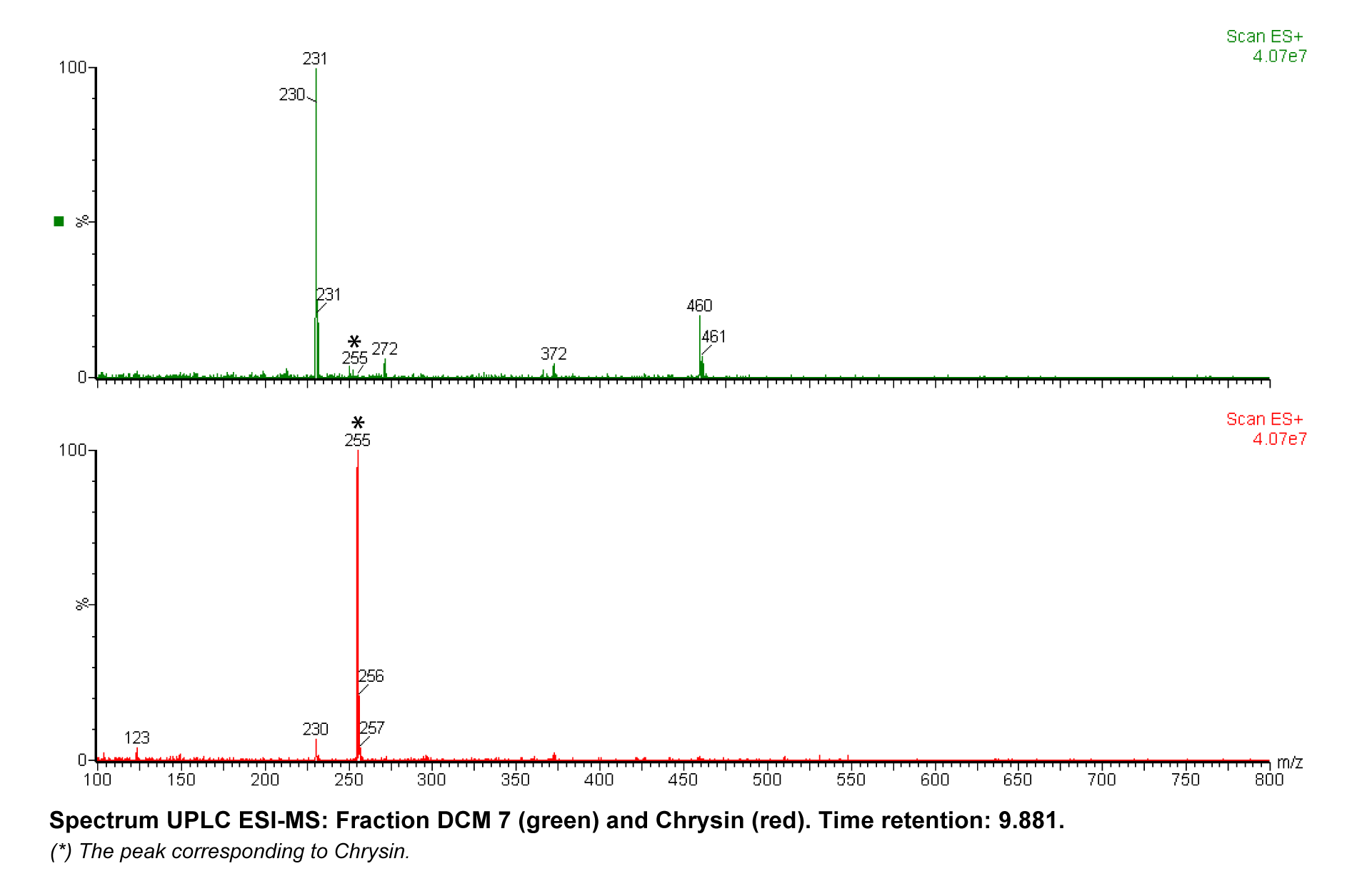

Supplement: Supplementary file 1 [file plants-09-00438-s001.zip › fraction dcm 7 and standard_Spectrum Fraction DCM 7 and Chrysin.tif]

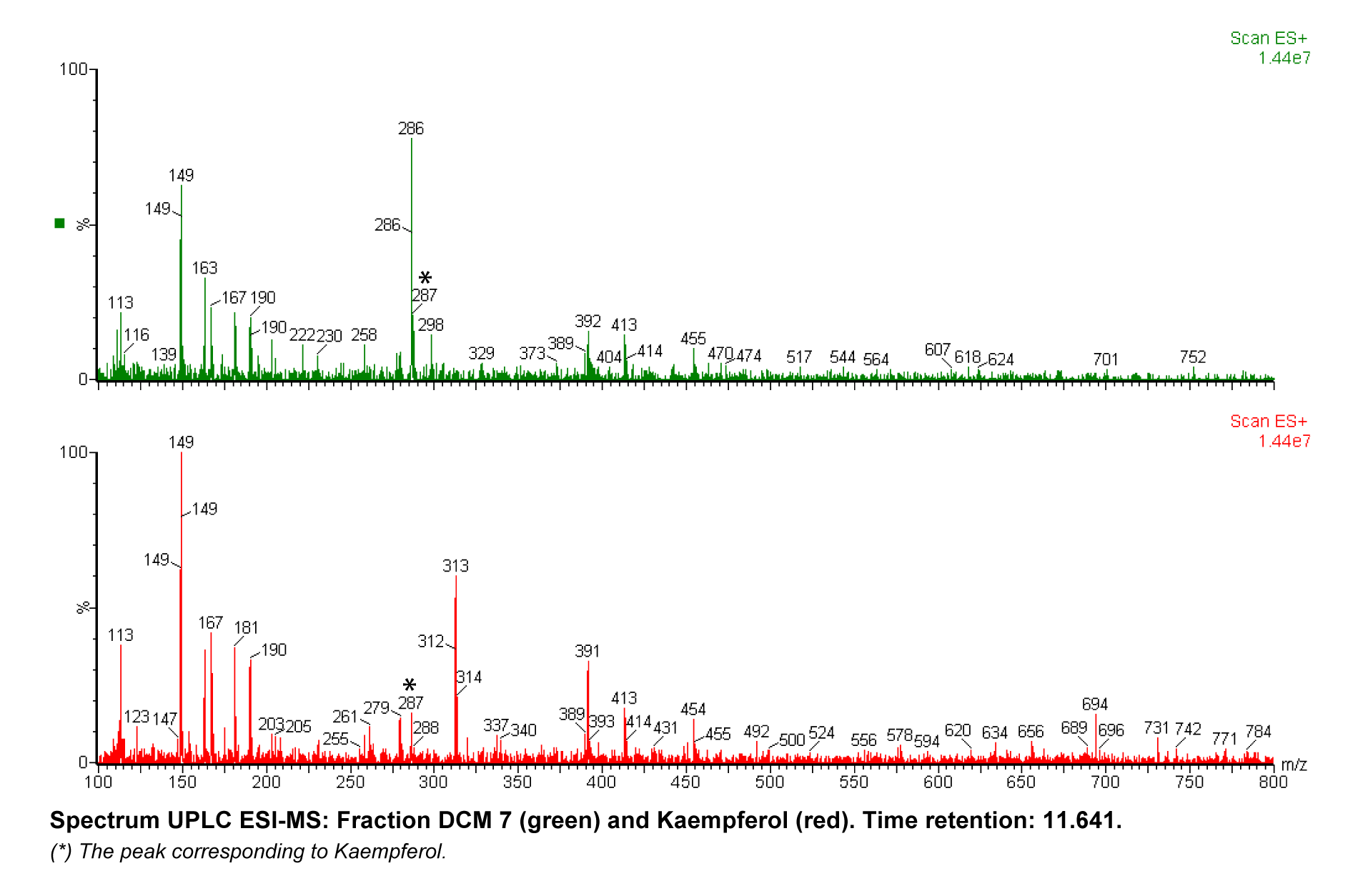

Supplement: Supplementary file 1 [file plants-09-00438-s001.zip › fraction dcm 7 and standard_Spectrum Fraction DCM 7 and Kaempferol.tif]

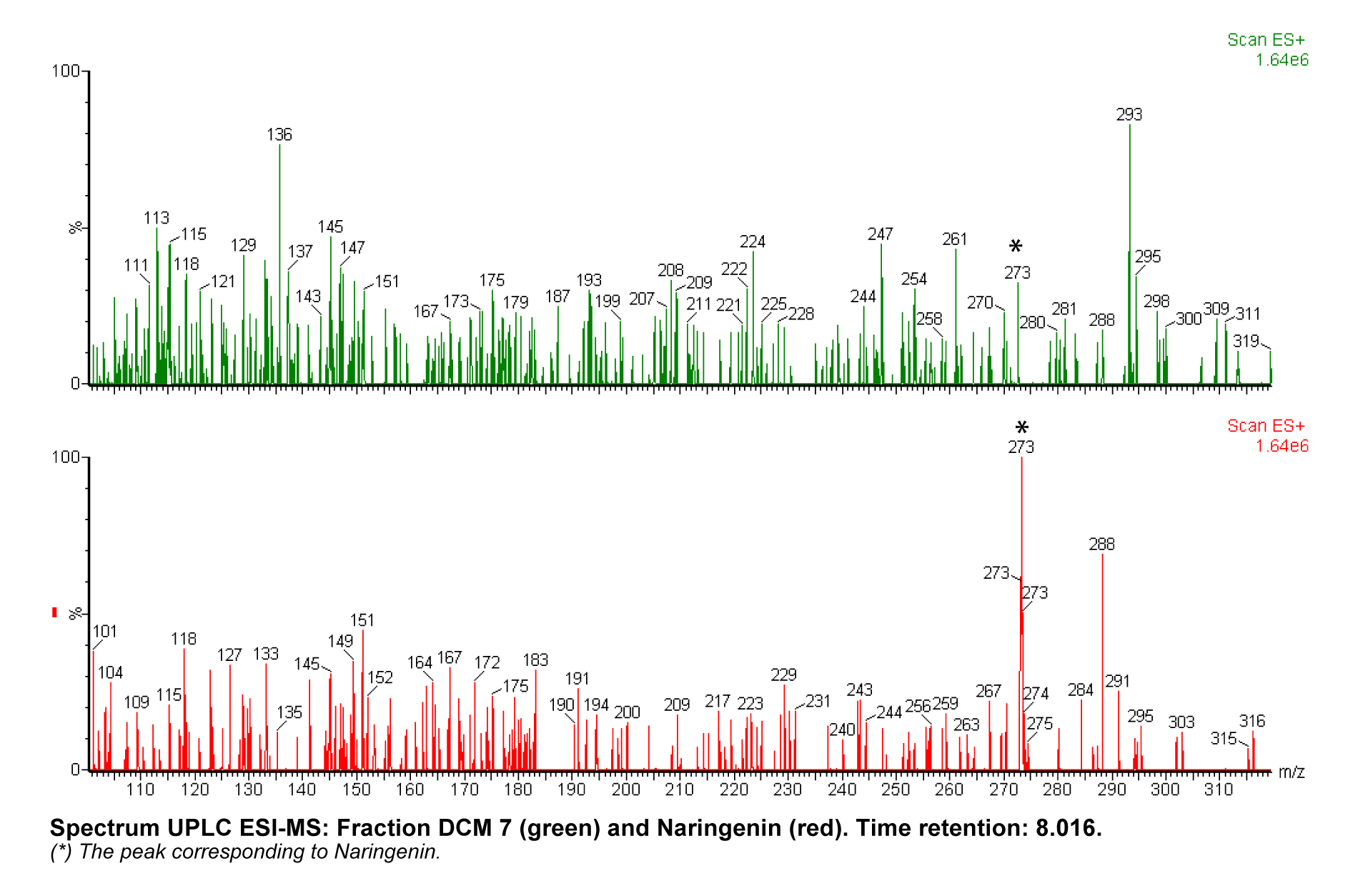

Supplement: Supplementary file 1 [file plants-09-00438-s001.zip › fraction dcm 7 and standard_Spectrum Fraction DCM 7 and Naringenin.tif]

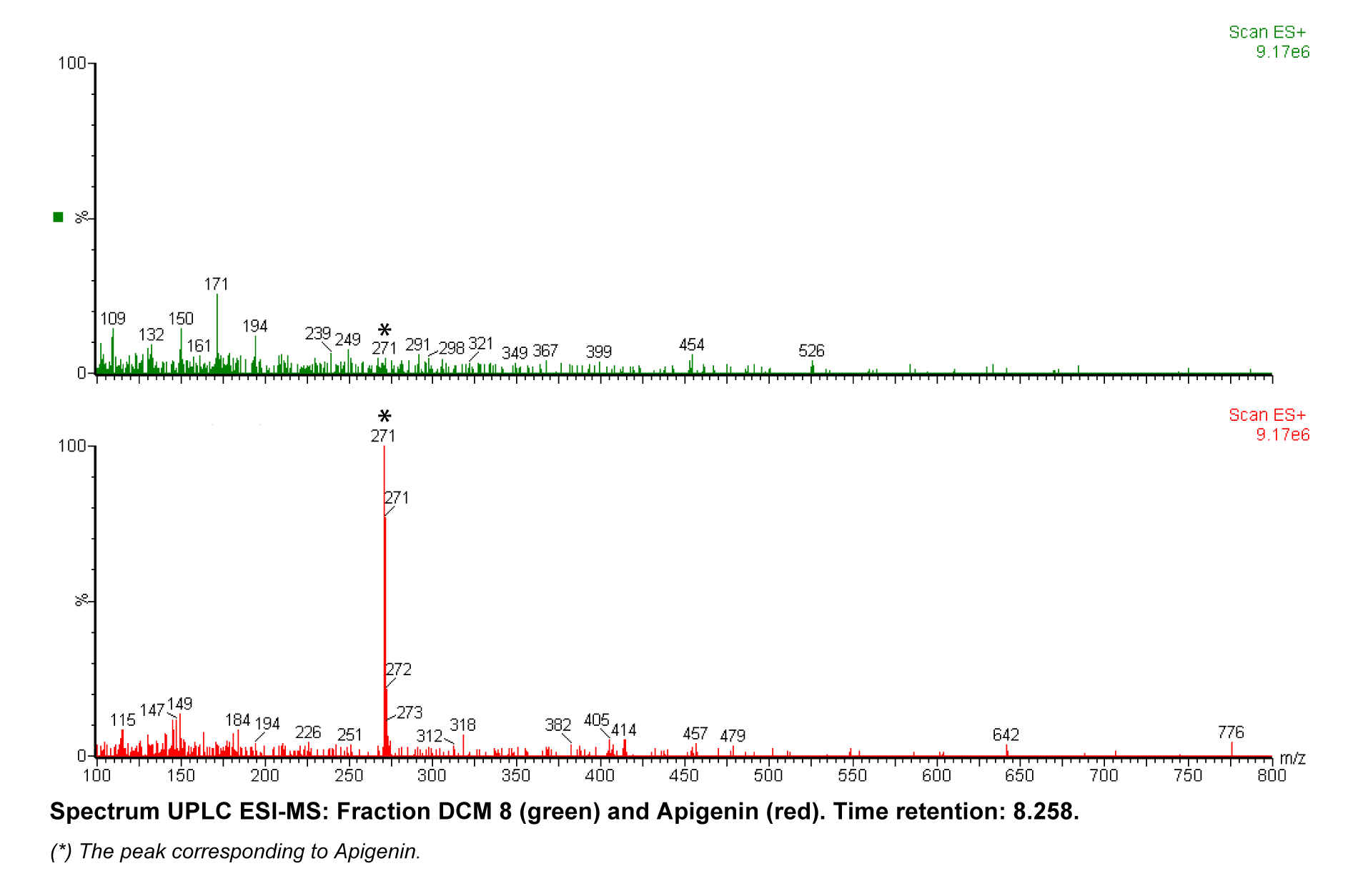

Supplement: Supplementary file 1 [file plants-09-00438-s001.zip › fraction dcm 8 and standard_Spectrum Fraction DCM 8 and Apigenin.tif]
